# The association of lipid accumulation product with inflammatory parameters and mortality: evidence from a large population-based study

**DOI:** 10.3389/fepid.2024.1503261

**Published:** 2025-02-04

**Authors:** Yi Chi, Yiqing Zhang, Huang Lin, Shanshan Zhou, Genlin Jia, Wei Wen

**Affiliations:** ^1^ Department of Integrative Medicine (Geriatrics), The People’s Hospital Medical Group of Xiangzhou, Zhuhai, China; ^2^ Department of Spleen and Gastroenteritis, Heilongjiang University of Chinese Medicine, Harbin, China; ^3^ Department of Cardiovascular Disease, Xiyuan Hospital of China Academy of Chinese Medical Sciences, Beijing, China

**Keywords:** abdominal volume index, all-cause mortality, cardiovascular mortality, inflammatory parameters, nomogram

## Abstract

**Background:**

Obesity is closely associated with lipid metabolism, and the accumulation of lipids leads to low-level inflammation in the body, which can trigger cardiovascular disease. This study aimed to explore the association between a novel marker of lipid accumulation, the abdominal volume index (AVI), inflammatory parameters, and mortality.

**Methods:**

This study enrolled 2,109 older adult senior citizens (aged over 60 years) with hypertension from the National Health and Nutrition Examination Survey. The primary endpoints included all-cause mortality and cardiovascular mortality, which were assessed by linking the data to the National Death Index records. Cox regression model and subgroup analysis were constructed to investigate the associations between AVI and both all-cause and cardiovascular mortality. Restricted cubic splines were employed to further explore the relationships among AVI, inflammatory parameters, and mortality. By considering inflammatory factors as mediators, we investigate the mediating effects of AVI on mortality.

**Results:**

After a median follow-up of 69 months, there were 1,260 deaths, with 337 attributed to cardiovascular causes within the older adult population studied. In the multivariable-adjusted model, AVI was positively associated with both all-cause and cardiovascular mortality [Hazard Ratio (HR) = 1.09, 95% CI = 1.06–1.11 for all-cause mortality; HR = 1.07, 95% CI = 1.03–1.12 for cardiovascular mortality]. Kaplan-Meier survival plots indicated an overall median survival time of 144 months. Mediation analysis revealed that Systemic Inflammatory Response Index (SIRI), Monocyte-to-HDL ratio (MHR), and Neutrophil-to-Lymphocyte ratio (NLR) mediated 27.15%, 35.15%, and 16.55%, respectively, of the association between AVI and all-cause mortality.

**Conclusion:**

AVI is positively associated with all-cause mortality in older adults with hypertension, and this association appears to be partially mediated by inflammatory parameters.

## Introduction

As the global population ages, the mortality associated with cardiovascular disease (CVD) continues to rise, presenting a significant public health challenge (1). CVD remains the leading cause of death among older adults, with nearly half of all cardiovascular deaths attributable to high blood pressure (2). The prevalence of hypertensive (defined as blood pressure ≥140/90 mmHg) has increased from 5.1% in 1958–1959 to 23.2% in 2012–2015 (3, 4). Cohort studies have demonstrated that hypertension is linked to a higher risk of all-cause mortality among the oldest segments of the population in China, showing a U-shaped association between systolic blood pressure and mortality (5), which indicates a need for increased healthcare resources to address the needs of hypertensive older adult individuals.

Obesity is significantly associated with increased all-cause mortality (6). It is characterized by excessive lipid accumulation and often accompanies metabolic abnormalities that trigger insulin resistance and related complications, including diabetes, CVD, and metabolic syndrome (MetS) (7, 8). There is growing concern over the adverse outcomes inked to central obesity, particularly abdominal fat. Although computed tomography (CT) and magnetic resonance imaging (MRI) are considered the gold standards for assessing abdominal fat distribution (9), commonly used anthropometric measures such as body mass index (BMI), waist circumference, and waist-to-hip ratio also provide valuable evaluations (10). The abdominal volume index (AVI), a novel parameter, measures overall abdominal volume and assesses fat distribution. Research indicates that AVI may be a superior predictor of MetS, particularly in women (11). This study aims to investigate the association between AVI and mortality and to further analyze whether this association is mediated through the inflammatory response.

## Methods

### Data source

National Health and Nutrition Examination Survey (NHANES) is a population-based representative survey performed by the National Center for Health Statistics (NCHS) of the Centers for Disease Control and Prevention (CDC) (12). Its primary goal is to assess the health status and disease risk factors in the American population (13). NHANES collects comprehensive information, including demographic data, disease-related questionnaire responses, examination results, and laboratory data, through structured home interviews, physical examinations performed at mobile centers, and laboratory tests conducted at analytical facilities. The datasets analyzed in this study can be accessed through the NHANES website (https://www.cdc.gov/nchs/nhanes/index.htm). The NHANES protocol was approved by the research ethics review board of NCHS, and informed consent was obtained from all participants (14).

In brief, we analyzed 6 cycles of NHANES data spanning 11 years, from 2007 to 2018 (2007–2008, 2009–2010, 2011–2012, 2013–2014, 2015–2016, 2017–2018). A total of 59,842 participants were identified from the NHANES 2007–2018 data. We excluded individuals under the age of 60 (*n* = 47,932) and those without hypertension (*n* = 4,585). After further excluding participants with missing data on all-cause mortality (*n* = 14), BMI (*n* = 157), waist circumference (*n* = 420), triglycerides (*n* = 152), complete blood count (*n* = 672), medical history (*n* = 458), the final analysis included 5,433 individuals (see Supplementary Documents for screening process plots).

### Mortality

The NHANES public-use linked mortality file as of December 31, 2018, correlated with NCHS with the National Death Index, was used to determine mortality status in the follow-up individuals (15). The main outcomes in this study were all-cause mortality and cardiovascular mortality.

### Definition of parameters

The parameters involving AVI, SIRI, NLR, and MHR were calculated using the below formulas:

The SIRI (systemic inflammatory response index) was defined as a neutrophil count × monocyte count/lymphocyte count and expressed as × 10^9 cells/µl (16).

The NLR (neutrophil-to-lymphocyte ratio) was defined as neutrophil count/lymphocyte count (17).

The MHR (monocyte to high-density lipoprotein) was defined as monocyte count/high-density lipoprotein (18, 19).

$$\mathrm{AVI}=\frac{2\times (WC_{\left(\mathrm{cm}\right)}{)}^{2}+0.7\times (WC_{\left(\mathrm{cm}\right)}-HC_{\left(\mathrm{cm}\right)}{)}^{2}}{1000}$$

*WC* waist circumference, *HC* height circumference. *CM* centimeter (the unit).

### Assessment of covariates

We downloaded various data from NHANES, including demographic information [age, gender, race, education levels, family income-to-poverty ratio (PIR)], smoking status, alcohol consumption, and medical history [heart failure (HF), coronary artery disease (CAD), angina, stroke, diabetes, and hypercholesterolemia]. At a mobile examination center, participants' waist circumference (WC), hip circumference (HC), and body mass index (BMI) were measured. BMI was calculated as the ratio of weight (kg) to height (cm) squared. Additionally, Serum specimens were collected as part of the NHANES laboratory examination component for 2007–2018, processed, stored, and sent to Collaborative Laboratory Services for analysis. At baseline, we measured complete blood count, plasma albumin (g/L), blood urea nitrogen (mg/dl), creatinine (µmol/L), uric acid (UA) (µmol/L), alanine aminotransferase (AST) (U/L), aspartate aminotransferase (U/L), glycohemoglobin (%), glucose (mmol/L), cholesterol (mmol/L), HDL-cholesterol (mmol/L), and triglyceride (mmol/L) levels. Detailed information regarding these methods is publicly available on the NHANES website (20). Race was classified into categories including Mexican American, other Hispanic, non-Hispanic White, non-Hispanic Black, or another race (21). Education level was categorized into three groups: less than high school, high school, and higher than high school. The family income-to-poverty ratio (PIR) was divided into three categories: less than 1.3 for low-income, between 1.3 and 3.5 for middle-income, and above 3.5 for high-income households (22). Smoker was defined as participants who had smoked more than 100 cigarettes in their lifetime (23), regardless of whether they had quit smoking at the time of the interview (24). Alcohol drinkers were defined as individuals who consumed at least 12 drinks in the year preceding the survey (25). Questionnaires were used to diagnose hypertension (BPD035), heart failure (MCQ160b), CAD (MCQ160c), angina (MCQ160d), heart attack (MCQ160e), and stroke (MCQ160f) (26).

### Statistical analysis

All data were analyzed using R statistics (version 4.2.2, https://cran.r-project.org/). The Kolmogorov–Smirnov test indicated that all continuous variables did not follow a normal distribution; therefore, these variables are presented as medians with interquartile ranges. Categorical variables are expressed as frequencies and percentages. To examine differences in variables across different levels of the Atrial Volume Index (AVI), we categorized AVI into tertiles, from the lowest to the highest. The Chi-squared test or the Kruskal-Wallis *H*-test was utilized to analyze differences in AVI across these four categories. The one-way ANOVA test, Kruskal-Wallis *H*-test, or Chi-squared test were employed to compare continuous or categorical variables within the tertile groups of AVI (27). A *p*-value of <0.05 was considered statistically significant.

Multivariate Cox regression models were used to estimate the associations between AVI and all-cause as well as cardiovascular mortality, calculating hazard ratios (HRs) and 95% confidence intervals (CIs). Model 1 was unadjusted, while Model 2 was adjusted for gender, age, race, and education level. Model 3 included further adjustments for poverty income ratio (PIR), body mass index (BMI), smoking status, alcohol consumption, diabetes, hypercholesterolemia, cholesterol, and triglycerides. Subgroup analyses were conducted to explore potential differences among specific populations based on gender, race, education, PIR, diabetes, coronary artery disease (CAD), angina, stroke, smoking, and drinking status. Kaplan-Meier curves were plotted to estimate survival probabilities over time across different AVI levels, with the log-rank test employed to assess disparities among the tertile curves. A restricted cubic spline (RCS) model was applied to investigate the non-linear relationships between AVI, inflammatory parameters, and all-cause or cardiovascular mortality. Additionally, multivariate logistic regression was conducted to assess the associations between inflammatory parameters and AVI. The receiver operating characteristic (ROC) curve was utilized to determine the cutoff value of AVI for identifying mortality. The same statistical methods were applied in multivariate Cox regression to explore the associations between inflammatory parameters and mortality. Subsequently, mediation analysis was performed to assess whether the relationship between AVI and mortality was partially mediated by inflammatory parameters. The presence of a mediating effect was defined by significant indirect, direct, and total effects (26).

Eventually, we developed a predictive model for AVI to assess mortality risk. To eliminate collinearity among variables, we employed the least absolute shrinkage and selection operator (LASSO) regression model to exclude certain variables (28). The model's evaluation was conducted using 10-fold cross-validation, and we plotted a curve concerning lambda values. A risk prediction nomogram plot was created based on key mortality-related variables, including AVI and inflammatory parameters (SIRI and MHR). The model's forecasting performance was quantified by the area under the curve (AUC) with a 95% confidence interval from the ROC curve. The clinical applicability of the model was assessed through decision curve analysis.

## Results

### Baseline characteristic

Table 1 presents the baseline characteristics of the individuals enrolled in this study across different AVI tertiles. Among the participants, the median AVI values were 23.93, with ranges from 19.91 in T1, 23.93 in T2, to 29.49 in T3. The median age of the 5,433 senior citizens was 70 years, with approximately 47.78% being male. The incidence of all-cause and cardiovascular mortality among these participants during a median follow-up of 69 months was 23.19% and 6.20%, respectively. Compared to those in the low AVI group, participants with elevated AVI levels exhibited a higher prevalence of smoking, alcohol consumption, lower education levels, and PIR. Additionally, in terms of medical history, participants in higher quartiles reported a greater prevalence of diabetes, hypercholesterolemia, heart failure (HF), CAD, angina, and heart attacks. Regarding physical examination metrics, the T3 group demonstrated significantly higher levels of weight, height, BMI, and WC. Participants with higher AVI levels also showed significantly elevated inflammatory parameters (including SIR, MHR, and NLR), complete blood cell counts (including leukocyte, lymphocyte, and monocyte counts), as well as higher levels of ALT, creatinine, uric acid (UA), glucose, triglycerides, and HbA1c.

**Table 1:** Characteristics of the study population.

Factors	Total (*n* = 5,433)	Tertiles of AVI			*p*-value
		T1 (*n* = 1,811)	T2 (*n* = 1,811)	T3 (*n* = 1,811)	
Characteristic bassline					
Gender					<0.01
Male, *n* (%)	2,596 (47.78)	503 (27.77)	941 (51.96)	1,152 (63.61)	
Female, *n* (%)	2,837 (52.22)	1,308 (72.23)	870 (48.04)	659 (36.39)	
Age, years	70.00 [64.00, 77.00]	71.00 [65.00,79.00]	70.00 [64.00,77.00]	68.00 [63.00,75.00]	<0.01
AVI	23.93 [21.15, 27.44]	19.91 [18.40,21.15]	23.93 [22.97,24.91]	29.49 [27.44,32.59]	<0.01
Education level, *n* (%)					0.05
Below high school	1,617 (29.76)	563 (31.09)	560 (30.92)	494 (27.28)	
High school	1,358 (25.00)	457 (25.23)	448 (24.74)	453 (25.01)	
Above high	2,458 (45.24)	791 (43.68)	803 (44.34)	864 (47.71)	
Race, *n* (%)					<0.01
Mexican American	629 (11.58)	193 (10.66)	249 (13.75)	187 (10.33)	
Other Hispanic	527 (9.70)	200 (11.04)	193 (10.66)	134 (7.40)	
Non-Hispanic White	2,564 (47.19)	740 (40.86)	806 (44.51)	1,018 (56.21)	
Non-Hispanic Black	1,257 (23.14)	399 (22.03)	449 (24.79)	409 (22.58)	
Other Race	456 (8.39)	279 (15.41)	114 (6.29)	63 (3.48)	
PIR, *n* (%)					0.107
<1.29	1,646 (30.30)	577 (31.86)	540 (29.82)	529 (29.21)	
1.30-3.49	2,277 (41.91)	770 (42.52)	758 (41.86)	749 (41.36)	
>3.50	1,510 (27.79)	464 (25.62)	513 (28.33)	533 (29.43)	
Outcomes					
All-cause mortality, *n* (%)					<0.01
YES	1,260 (23.19)	382 (21.09)	385 (21.26)	493 (27.22)	
NO	4,173 (76.81)	1,429 (78.91)	1,426 (78.74)	1,318 (72.78)	
Cardiovascular mortality, *n* (%)					<0.01
YES	337 (6.20)	99 (5.47)	99 (5.47)	139 (7.68)	
NO	5,096 (93.80)	1,712 (94.53)	1,712 (94.53)	1,672 (92.32)	
Follow-up time (months)	69.00 [37.00, 108.00]	72.00 [38.00,108.00]	71.00 [38.00,108.50]	67.00 [36.00,105.00]	0.181
Medical history					
Drinking, *n* (%)	3,726 (68.58)	1,101 (60.80)	1,251 (69.08)	1,374 (75.87)	<0.01
Smoking, *n* (%)	2,753 (50.67)	728 (40.20)	946 (52.24)	1,079 (59.58)	<0.01
Diabetes, *n* (%)	1,854 (34.12)	426 (23.52)	614 (33.90)	814 (44.95)	<0.01
Hypercholesterolemia, *n* (%)	3,478 (64.02)	1,100 (60.74)	1,209 (66.76)	1,169 (64.55)	<0.01
HF, *n* (%)	489 (9.00)	107 (5.91)	145 (8.01)	237 (13.09)	<0.01
CAD, *n* (%)	701 (12.90)	167 (9.22)	249 (13.75)	285 (15.74)	<0.01
Angina, *n* (%)	381 (7.01)	91 (5.02)	132 (7.29)	158 (8.72)	<0.01
Heart attack, *n* (%)	630 (11.60)	151 (8.34)	213 (11.76)	266 (14.69)	<0.01
Stroke, *n* (%)	553 (10.18)	188 (10.38)	185 (10.22)	180 (9.94)	0.906
Physical examination					
Weight (kg)	78.60 [67.70, 91.70]	63.80 [57.60,68.95]	78.60 [73.60,83.90]	98.10 [89.95,109.05]	<0.01
Height (cm)	164.40 [157.30, 171.90]	158.80 [153.50,164.70]	165.60 [158.70,171.50]	170.30 [162.50,177.00]	<0.01
BMI (kg/m^2^)	28.90 [25.70, 33.00]	24.90 [22.70,27.10]	28.70 [26.70,31.14]	34.20 [31.16,38.40]	<0.01
WC (cm)	102.80 [94.30, 112.20]	90.70 [85.20,94.70]	102.90 [100.00,105.70]	117.20 [112.20,124.70]	<0.01
Inflammatory indexes					
SIRI,	1.03 [0.69,1.58]	1.19 [0.80,1.80]	1.31 [0.89,1.97]	1.03 [0.69,1.58]	<0.01
MHR	0.35 [0.25,0.48]	0.44 [0.32,0.59]	0.50 [0.37,0.66]	0.35 [0.25,0.48]	<0.01
NLR	2.00 [1.44,2.80]	2.09 [1.56,2.92]	2.23 [1.62,3.00]	2.00 [1.44,2.80]	<0.01
Blood cell count					
Leukocyte, 10^9^/L	6.90 [5.70, 8.30]	6.50 [5.40,7.80]	6.90 [5.70,8.20]	7.30 [6.10,8.70]	<0.01
Lymphocyte, 10^9^/L	1.90 [1.50, 2.40]	1.80 [1.50,2.40]	1.90 [1.50,2.40]	2.00 [1.50,2.50]	<0.01
Monocyte, 10^9^/L	0.60 [0.40, 0.70]	0.50 [0.40,0.60]	0.60 [0.50,0.70]	0.60 [0.50,0.70]	<0.01
Neutrophils, 10^9^/L	4.00 [3.20, 5.20]	3.70 [2.90,4.70]	4.00 [3.20,5.10]	4.40 [3.40,5.50]	<0.01
Platelet, 10^9^/L	229.00 [191.00, 272.00]	233.00 [196.00,278.00]	228.00 [191.00,271.00]	226.00 [186.00,268.00]	<0.01
Laboratory examination					
ALT (U/L)	21.00 [17.00, 26.00]	20.00 [16.00,25.00]	21.00 [17.00,26.00]	22.00 (17.00,27.00]	<0.01
AST (U/L)	22.00 [18.00, 27.00]	22.00 [18.00,26.00]	22.00 [18.00,27.00]	22.00 [18.00,28.00]	0.042
Albumin (g/L)	42.00 [40.00, 44.00]	42.00 [40.00,44.00]	42.00 [40.00,44.00]	41.00 [39.00,43.00]	<0.01
Bun (mg/ml)	16.00 [13.00, 20.00]	16.00 [12.00,20.00]	16.00 [13.00,20.00]	16.00 [13.00,21.00]	<0.01
Creatinine (umol/L)	83.98 [69.84, 102.54]	78.68 [64.53,95.47]	83.98 [70.72,103.43]	88.40 [73.37,106.96]	<0.01
UA (umol/L)	345.00 [291.50, 404.50]	315.20 [261.70,368.80]	345.00 [291.50,410.40]	368.80 [315.20,428.30]	<0.01
Glucose (mmol/L)	5.61 [5.05, 6.66]	5.38 [4.94,6.11]	5.61 [5.05,6.66]	5.94 [5.22,7.27]	<0.01
Cholesterol (mmol/L)	4.76 [4.03, 5.53]	4.99 [4.27,5.71]	4.73 [4.03,5.53]	4.50 [3.85,5.33]	<0.01
Triglyceride (mmol/L)	1.51 [1.03, 2.22]	1.32 [0.90,1.94]	1.52 [1.04,2.22]	1.69 [1.19,2.46]	<0.01
HDL (mmol/L)	1.32 [1.09, 1.63]	1.50 [1.24,1.81]	1.29 [1.08,1.58]	1.19 [1.01,1.40]	<0.01
HbA1c (%)	5.90 [5.60, 6.40]	5.80 [5.50,6.10]	5.90 [5.50,6.40]	6.10 [5.70,6.80]	<0.01

WC, waist circumference; AVI, abdominal volume index; PIR, poverty-to-income ratio; BMI, body mass index; NLR, neutrophil to lymphocyte ratio; SIRI, systemic inflammatory response index; MHR, monocyte to high density lipoprotein; CAD, coronary artery disease; HF, heart failure; HDL, high-density lipoprotein cholesterol; BUN, blood urea nitrogen; UA, uric acid; ALT, alanine aminotransferase; AST, aspartate aminotransferase; HBA1c, hemoglobin A1c.

### Associations of AVI with all-cause and cardiovascular mortality

**Figure 1:**
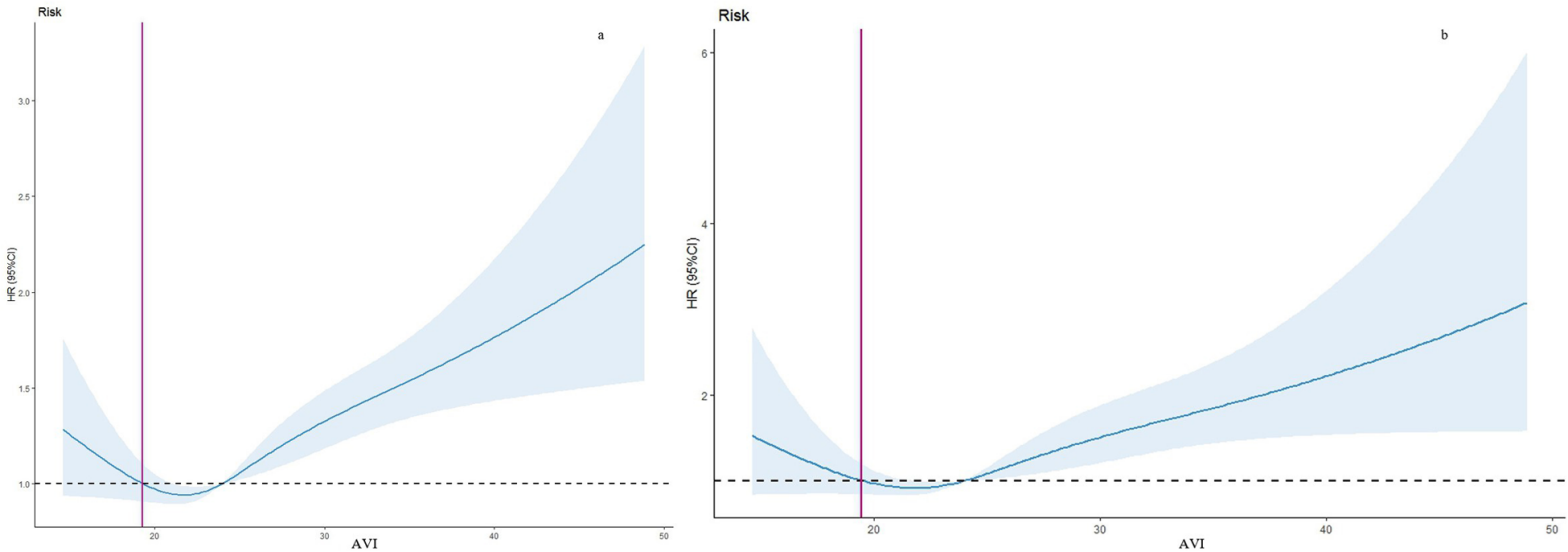
Kaplan–Meier curves of the survival rate of participants with abdominal volume index (AVI) tertiles. **(a)** All-cause mortality. **(b)** Cardiovascular mortality.

In this study, after a median follow-up of 69 months, we observed 1,260 deaths, including 337 attributed to cardiovascular causes, within the older adult population. Table 2 illustrates the associations between the Atherogenic Index of Plasma (AVI) and both all-cause and cardiovascular mortality. In the crude model, AVI demonstrated a significant association with an increased risk of all-cause mortality (HR = 1.03, 95% CI = 1.02–1.04) and cardiovascular mortality (HR = 1.04, 95% CI = 1.02–1.06). These associations remained robust and statistically significant after multivariable adjustments, with Model 2 showing HRs of 1.04 (95% CI = 1.03–1.05) for all-cause mortality and 1.06 (95% CI = 1.03–1.08) for cardiovascular mortality, and Model 3 showing HRs of 1.09 (95% CI = 1.06–1.11) and 1.07 (95% CI = 1.03–1.12) respectively. Compared to the first tertile of AVI, the hazard ratios for participants in the second, third, and fourth tertiles were consistently higher, regardless of variable adjustments. The Kaplan-Meier survival plots presented in Figure 1 indicate significant differences in all-cause and cardiovascular mortality across AVI tertiles (*P* < 0.01), with an overall median survival time of 144 months in tertile 3 for all-cause mortality.

**Table 2:** The associations of AVI with all-cause mortality and cardiovascular mortality.

	HR (95% CI)		
	Model 1	Model 2	Model 3
All-cause mortality			
AVI	1.03 (1.02, 1.04)**	1.04 (1.03, 1.05)**	1.09 (1.06,1.11)**
Tertiles 1	1	1	1
Tertiles 2	1.01 (0.88, 1.16)	0.99 (0.86, 1.15)	0.98 (0.83, 1.15)
Tertiles 3	1.34 (1.17, 1.53)**	1.38 (1.19, 1.59)**	1.36 (1.10, 1.68)**
Cardiovascular mortality			
AVI	1.04 (1.02, 1.06)**	1.06 (1.03, 1.08)**	1.07 (1.03, 1.12)**
Tertiles 1	1	1	1
Tertiles 2	1.00 (0.76, 1.32)	0.97 (0.73, 1.29)	0.85 (0.63, 1.16)
Tertiles 3	1.46 (1.13, 1.89)**	1.48 (1.12, 1.96)**	1.10 (0.74, 1.64)

Model 1 was unadjusted.

Model 2 was adjusted for gender, age, race, and education level.

Model 3 was additionally modified to account for PIR, BMI, smoking, drinking, diabetes, hypercholesterolemia, cholesterol, triglycerides.

AVI, abdominal volume index; BMI, body mass index; PIR, Ratio of family income to poverty; HR, hazard ratio; CI, confidence interval.

*P < 0.01.

**P < 0.05.

### Results of nonlinear AVI and mortality

**Figure 2:**
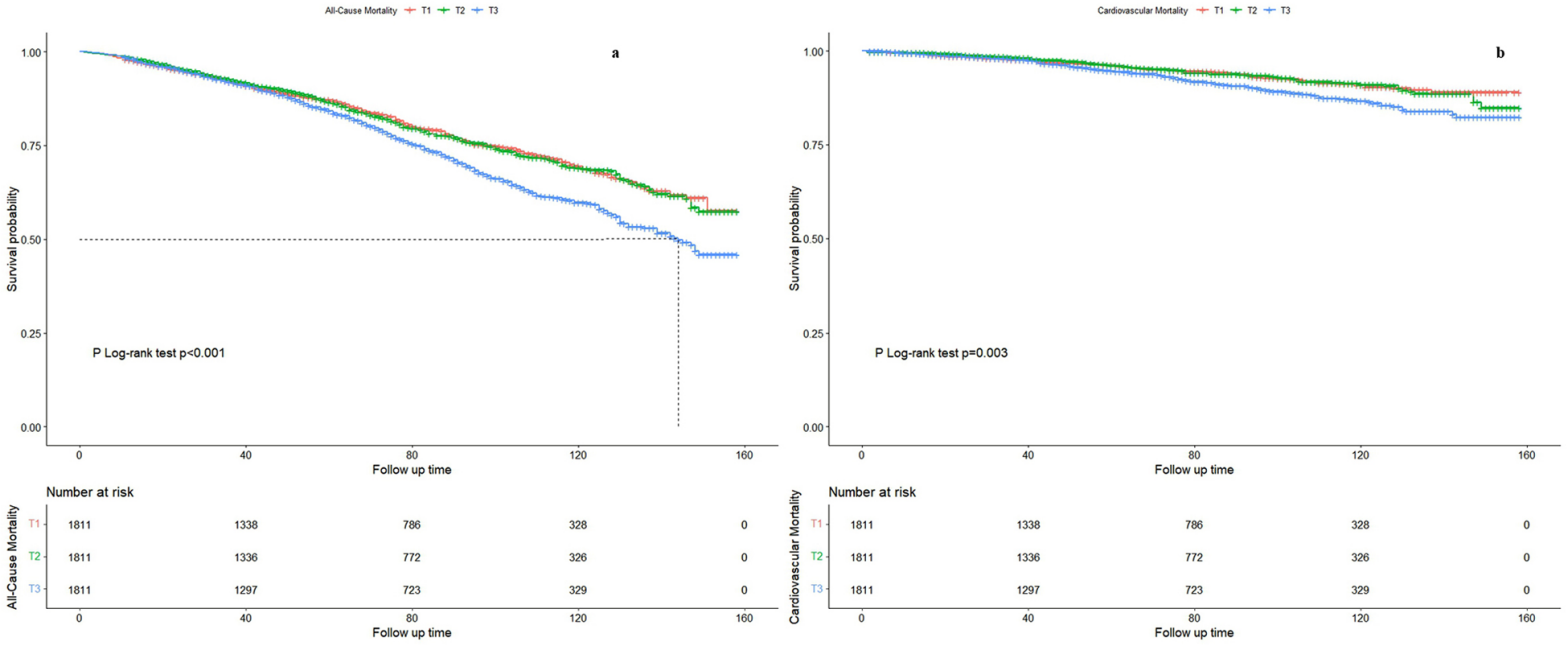
Restricted cubic spline regression curve of the association between abdominal volume index (AVI) and mortality. **(a)** All-cause mortality. **(b)** Cardiovascular mortality. The value corresponding to the red vertical line is the cutoff value in the *x*-axis. *HR,* hazard ratio; *CI,* confidence interval.

Using restricted cubic spline regression (RCS) models adjusted for the aforementioned confounders, we identified an L-shaped association between Dietary Inflammatory Index (DII) and both all-cause and cardiovascular mortality (Figure 2). The cut-off values were determined to be 19.26 for all-cause mortality and 19.43 for cardiovascular mortality.

### Subgroup analysis

**Figure 3:**
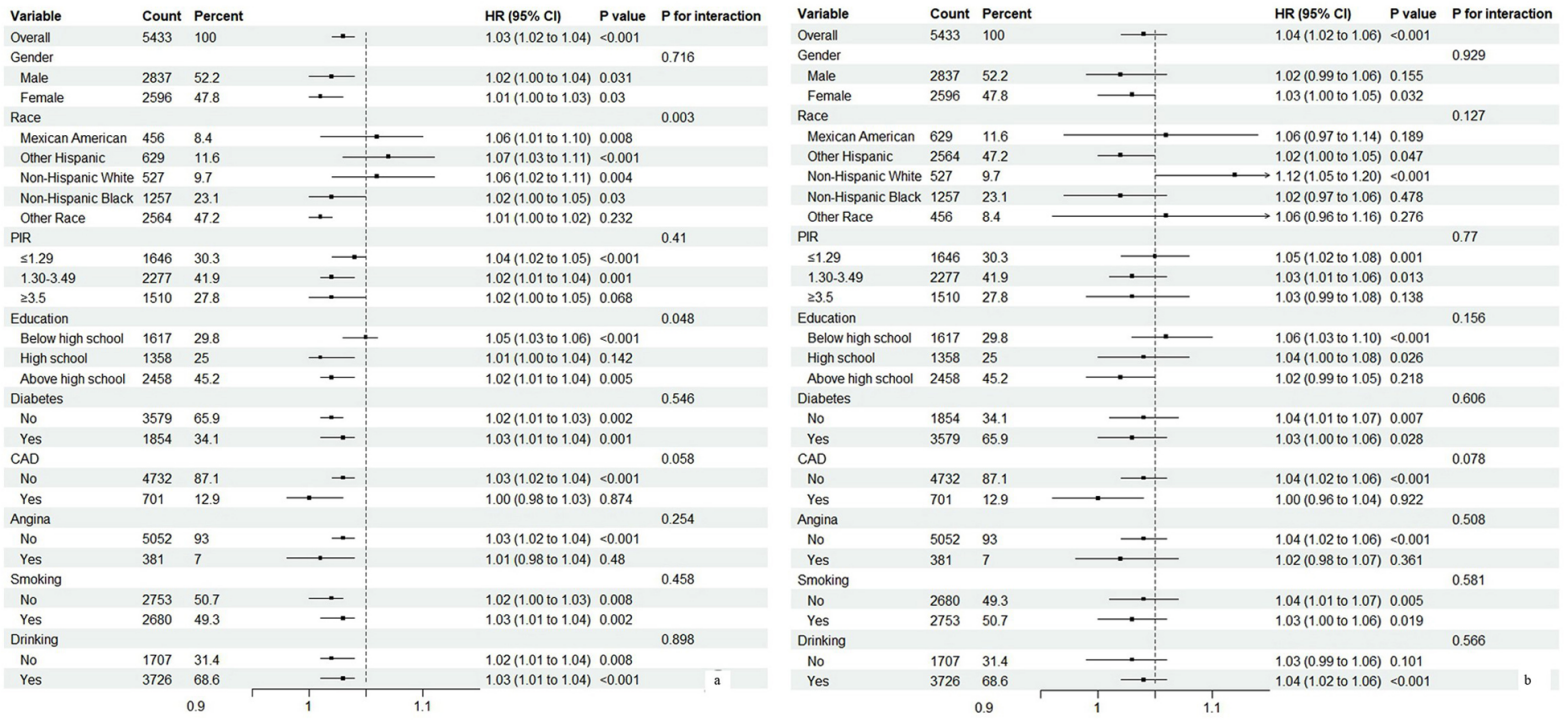
Subgroup analysis of associations between abdominal volume index (AVI) and mortality. **(a)** All-cause mortality. **(b)** Cardiovascular mortality.

We conducted subgroup analyses to further explore the relationships between AVI and mortality, with detailed results presented in Figure 3. Most analyses revealed differences within groups; however, the association between AVI and all-cause mortality was not significant among participants of other races, those with a PIR ≥ 3.5, individuals with only a high school diploma, and those with coronary artery disease (CAD) or angina. For cardiovascular mortality, significant differences were mostly absent across subgroup analyses, except for female participants, Hispanic participants, those with a PIR < 3.5, individuals with a high school education or less, and non-diabetics who did not have CAD, angina, or a smoking and drinking history.

### Associations of inflammatory parameters with AVI

Table 3 displays the associations between AVI and inflammatory parameters as determined by multivariate logistic regression. The results indicate that AVI is positively associated with Systemic Inflammation Response Index (SIRI) (OR = 1.12, 95% CI = 1.07–1.18), Monocyte to High-Density Lipoprotein Ratio (MHR) (OR = 5.69, 95% CI = 4.47–7.24), and Neutrophil to Lymphocyte Ratio (NLR) (OR = 1.04, 95% CI = 1.01–1.08). We employed RCS models with three knots (10th, 50th, and 90th percentiles) to assess the nonlinearity of these associations, confirming an L-shaped relationship (see Supplementary Documents).

**Table 3:** The associations between AVI and inflammatory parameters.

	OR	95% CI	*P*-value
SIRI			
Model 1	1.12	1.07, 1.18	<0.01
Model 2	1.08	1.02, 1.13	<0.01
Model 3	1.05	0.98, 1.13	=0.151
MHR			
Model 1	5.69	4.47, 7.24	<0.01
Model 2	3.72	2.90, 4.76	<0.01
Model 3	1.58	1.15, 2.17	<0.01
NLR			
Model 1	1.04	1.01, 1.08	=0.016
Model 2	1.02	0.99, 1.06	=0.23
Model 3	1.05	0.99, 1.11	=0.092

Model 1 was unadjusted.

Model 2 was adjusted for gender, age, race, and education level.

Model 3 was additionally modified to account for PIR, BMI, smoking, drinking, diabetes, hypercholesterolemia, cholesterol, triglycerides.

NLR, neutrophil to lymphocyte ratio; SIRI, systemic inflammatory response index; MHR, monocyte to high density lipoprotein; AVI, abdominal volume index; BMI, body mass index; PIR, ratio of family income to poverty; OR, odds ratio; CI, confidence interval.

### Associations of inflammatory parameters and mortality

Cox regression results examining the impact of inflammatory parameters on all-cause and cardiovascular mortality are summarized in Table 4. All indicators were positively associated with all-cause mortality, and most factors also correlated with cardiovascular mortality, with the exception of MHR in Model 3. Additionally, RCS models adjusted for confounders revealed an anhaped association between the two (see supplementary documents.

**Table 4:** The associations of inflammatory parameters with all-cause mortality and cardiovascular mortality.

	HR (95% CI)		
	Model 1	Model 2	Model 3
All-cause mortality			
SIRI	1.23 (1.21, 1.25)	1.19 (1.16, 1.21)	1.19 (1.16, 1.22)
MHR	1.25 (1.18, 1.32)	1.16 (1.10, 1.24)	1.14 (1.07, 1.21)
NLR	1.14 (1.12, 1.15)	1.11 (1.09, 1.13)	1.11 (1.09, 1.13)
Cardiovascular mortality			
SIRI	1.24 (1.20, 1.28)	1.20 (1.15, 1.25)	1.20 (1.15, 1.25)
MHR	1.25 (1.12, 1.39)	1.14 (1.01, 1.29)*	1.12 (0.98, 1.27)&
NLR	1.15 (1.12, 1.18)	1.12 (1.08, 1.15)	1.12 (1.08, 1.16)

Model 1 was unadjusted.

Model 2 was adjusted for gender, age, race, and education level.

Model 3 was additionally modified to account for PIR, BMI, smoking, drinking, diabetes, hypercholesterolemia, cholesterol, triglycerides.

NLR, neutrophil to lymphocyte ratio; SIRI, systemic inflammatory response index; MHR, monocyte to high density lipoprotein; AVI, abdominal volume index; BMI, body mass index; PIR, Ratio of family income to poverty; HR, hazard ratio; CI, confidence interval.

*P < 0.01.

**P < 0.05.

### The mediating role of inflammatory parameters

**Figure 4:**
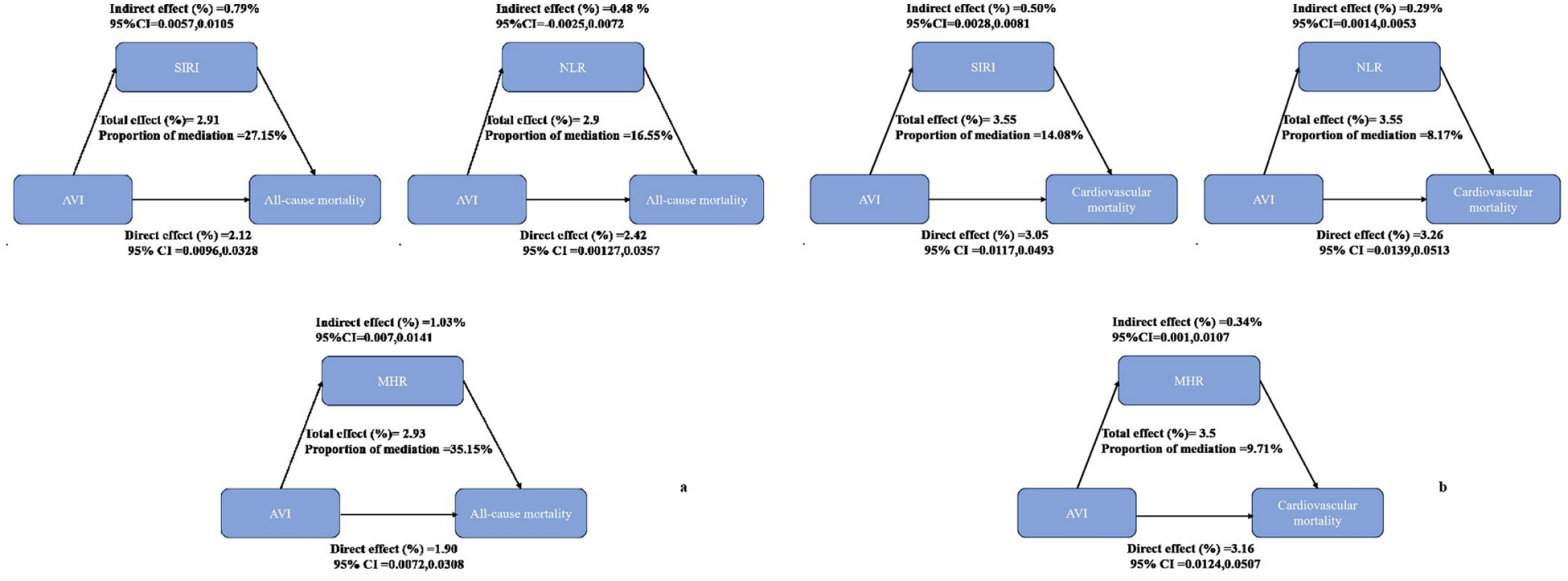
Analysis of the mediation by inflammatory parameters including systemic inflammatory response index (SIRI), neutrophil-to-lymphocyte ratio (NLR), and monocyte-to-high-density lipoprotein (MHR). **(a)** All-cause mortality. **(b)** Cardiovascular mortality.

Figure 4(a) illustrates the mediation effects of SIRI, MHR, and NLR on the relationship between AVI and all-cause mortality, accounting for 27.15%, 35.15%, and 16.55% of the association, respectively. Figure 4(b) shows that these parameters mediated 14.08%, 9.71%, and 8.17% of the association between AVI and cardiovascular mortality.

### Establishment of risk nomogram

We developed a risk prediction model utilizing LASSO penalized regression, incorporating 33 additional covariates (including age, gender, PIR, BMI, and laboratory examination results) to assess the impact of AVI on all-cause mortality. Through LASSO regression and a 10-fold cross-validation method for model evaluation, we identified 12 significant variables for inclusion in the nomogram. The predictive performance for AVI was validated using the ROC curve (AUC = 0.76 for the total), with performance metrics for 2, 3, and 4 years being 0.62, 0.56, and 0.52, respectively (see Supplementary Documents).

## Discussions

In this study, we identified a positive association between Abdominal Volume Index (AVI) and mortality among older adults with hypertension. Notably, this association remained significant even after applying a comprehensive adjustment model. Our findings demonstrated positive correlations among inflammatory parameters, mortality, and AVI, as indicated by Cox and logistic regression analyses. To further explore the potential inflammatory mechanisms linking AVI to mortality, we employed a mediation effect model to examine the significant roles of the Systemic Inflammation Response Index (SIRI), Monocyte to High-Density Lipoprotein Ratio (MHR), and Neutrophil to Lymphocyte Ratio (NLR). Given that both AVI and inflammatory parameters serve as risk factors for mortality, we developed a prognostic nomogram model, which was validated to effectively predict all-cause mortality.

Obesity, characterized by excessive body fat accumulation, has become a major global public health challenge. The prevalence of obesity in adults has risen dramatically, increasing from 3% in 1975 to 11% among men and from 6% to 15% among women by 2016 (29). A larger-scale study indicated that obesity throughout adulthood, as well as weight gain from youth to middle age, correlates with higher mortality risk (29). Defined as a chronic and complex disease, obesity can lead to various adverse cardiovascular events (30, 31). Body Mass Index (BMI) is a widely recognized indicator of obesity, with a well-established J-shaped association with mortality (32). However, many previous cohort studies relied on a single BMI measurement, neglecting other parameters that assess lipid accumulation. Indicators like AVI, which are easy to calculate and readily accessible, serve as proxies for visceral fat accumulation and are linked to impaired glucose tolerance and insulin resistance (IR). Fernando Guerrero-Romero highlighted AVI as a reliable tool for estimating overall abdominal volume, showing a strong relationship with IR (OR = 1.6, 95% CI: 1.1–9.1) (33). By better assessing abdominal fat accumulation, AVI offers improved predictions for the development of metabolic syndrome (MetS) (34). Given the interrelationship among IR, MetS, and diabetes, evaluating abdominal fat is crucial for assessing adiposity and predicting the risks of Type 2 Diabetes (T2D) and MetS (35). These metabolic diseases can impair vascular function, leading to atherosclerosis and cardiovascular events. A study found that AVI is an optimal predictor of cardiometabolic abnormalities in a cohort of Lebanese adults, with a cutoff value of 19.61 (36). Furthermore, abdominal fat deposition correlates with various cardiometabolic risk factors, including blood pressure, triglycerides, and cholesterol levels (37). A prospective cohort to investigate the impact of computed tomography (CT)-measured abdominal fat levels on mortality in undergoing hemodialysis patients, reported that high abdominal fat distribution has a high risk of death (38).

Moreover, numerous inflammatory parameters have been consistently linked to both obesity and the risk of adverse outcomes associated with obesity-related diseases (39). A meta-analysis encompassing 51 cross-sectional studies supports a positive correlation between C-reactive protein (CRP) and various obesity indicators, including BMI, waist circumference (WC), and waist-to-hip ratio (40). Increases in various inflammatory parameters have also been associated with an increased risk of obesity-related diseases, including cardiovascular disease (41, 42). Furthermore, several studies have indicated that elevated circulating levels of inflammatory cytokines, such as tumor necrosis factor (TNF)*α*, interleukin (IL)-6, or CRP, have been documented in overweight adults (43–45). Interestingly, other markers of abdominal obesity (e.g., WC) appear to be more strongly associated with inflammatory parameters than BMI (46), indicating a greater impact of central obesity on inflammation. Similarly, VAI as a novel marker of abdominal fat can be applied to characterize lipid accumulation, and our study confirms this perspective. Specifically, AVI and inflammatory parameters involving SIRI, MHR, and NLR exhibit an L-shaped association. Moreover, the relationship between lipid accumulation and inflammatory parameters has been described in previous studies. For instance, Hermsdorff revealed that indices of abdominal fat accumulation were associated with CRP, IL-6, and retinol-binding protein 4 concentrations (47). Even in non-obese subjects, CRP has a positive association with abdominal fat (48). Some studies have reported that adipocytes can secrete substantial bioactive molecules with immuno-modulatory actions, such as leptin and adiponectin (49, 50). Leptin can stimulate monocyte proliferation and differentiation into macrophages, which induces the production of pro-inflammatory cytokines such as TNF*α*, IL-6, or IL-12 (51). Elevated blood lipid levels in obese adults may produce a toxic effect on their adipose tissue according to the “adipose tissue expandability” hypothesis (52). Additionally, fatty acids can trigger the inflammatory response by modulating adipokine production or secretion. NEFA can cause an inflammatory response by modulating adipokine production or activating Toll-like receptors (53).

There is considerable evidence supporting the correlation between inflammation and mortality. Yiyuan Xia reported that adults with SIRI levels greater than 1.43 had a higher risk of all-cause mortality (HR: 1.39, 95%CI: 1.26–1.52) and cardiovascular death (HR: 1.39, 95%CI: 1.14–1.68) (54). This study also provides evidence for these novel inflammation indices in predicting mortality. Notably, inflammation indicators may correspond to various signaling pathways, such as mitogen-activated protein kinase (MAPK) pathways, PI3 K/Akt pathways, and NF-*κ*B signaling pathways (55). The PI3 K/Akt pathway can promote cell survival by inhibiting apoptotic processes through mediating responses to chemokines and other inflammatory stimuli (56). TNFR1 activates the MAPK and NF-*κ*B pathways to promote inflammation leading to apoptosis (57). In this study, we found that inflammatory parameters mediated the association between AVI and mortality. Xiaoqi Deng also indicated a significant association between SII and both all-cause and cardiovascular mortality (58).

This study was a prospective cohort study with an explicit causal association. We provided additional evidence supporting the positive associations of AVI with all-cause mortality in older adults with hypertension. We also highlight the mediating role of inflammation in the associations of AVI with mortality. In summary, we underscore that obesity may cause lipid accumulation, further triggering low-grade inflammation in the body and related poor prognosis of obesity. The main limitation of this study is that diagnoses of hypertension, DM, heart failure, etc., were determined by questionnaires, which may introduce recall bias, potentially affecting the accuracy of covariates.

## Conclusions

This study provided evidence for the positive associations of AVI with all-cause mortality in the older population with hypertension, while also highlighting the significant mediating role of inflammatory indices in this association.

## Data Availability

Publicly available datasets were analyzed in this study. This data can be found here: https://www.cdc.gov/nchs/nhanes/index.htm.
