# Supplementary material for: The association of lipid accumulation product with inflammatory parameters and mortality: evidence from a large population-based study
Source: Front Epidemiol. 2025 Feb 4;4:1503261. doi: 10.3389/fepid.2024.1503261 (PMC11832662; doi:10.3389/fepid.2024.1503261)
Supplement: Supplementary file 1 [file Supplementaryfile2.docx]

**Table1 Characteristics of the study population**

| **Factors** | **Total**  **(n=5433)** | **Tertiles of AVI** | | | **p value** |
| --- | --- | --- | --- | --- | --- |
|  |  | T1 (n=1811) | T2 (n=1811) | T3 (n =1811) |  |
| Characteristic bassline | | | | | |
| Gender | | | | | <0.01 |
| Male, n (%) | 2596 (47.78) | 503 (27.77) | 941 (51.96) | 1152 (63.61) |  |
| Female, n (%) | 2837 (52.22) | 1308 (72.23) | 870 (48.04) | 659 (36.39) |  |
| Age, years | 70.00 [64.00, 77.00] | 71.00 [65.00,79.00] | 70.00 [64.00,77.00] | 68.00 [63.00,75.00] | <0.01 |
| AVI | 23.93 [21.15, 27.44] | 19.91 [18.40,21.15] | 23.93 [22.97,24.91] | 29.49 [27.44,32.59] | <0.01 |
| Education level, n (%) | | | | | 0.05 |
| Below high school | 1617 (29.76) | 563 (31.09) | 560 (30.92) | 494 (27.28) |  |
| High school | 1358 (25.00) | 457 (25.23) | 448 (24.74) | 453 (25.01) |  |
| Above high | 2458 (45.24) | 791 (43.68) | 803 (44.34) | 864 (47.71) |  |
| Race, n (%) | | | | | <0.01 |
| Mexican American | 629 (11.58) | 193 (10.66) | 249 (13.75) | 187 (10.33) |  |
| Other Hispanic | 527 (9.70) | 200 (11.04) | 193 (10.66) | 134 (7.40) |  |
| Non-Hispanic White | 2564 (47.19) | 740 (40.86) | 806 (44.51) | 1018 (56.21) |  |
| Non-Hispanic Black | 1257 (23.14) | 399 (22.03) | 449 (24.79) | 409 (22.58) |  |
| Other Race | 456 (8.39) | 279 (15.41) | 114 (6.29) | 63 (3.48) |  |
| PIR, n (%) | | | | | 0.107 |
| <1.29 | 1646 (30.30) | 577 (31.86) | 540 (29.82) | 529 (29.21) |  |
| 1.30-3.49 | 2277 (41.91) | 770 (42.52) | 758 (41.86) | 749 (41.36) |  |
| >3.50 | 1510 (27.79) | 464 (25.62) | 513 (28.33) | 533 (29.43) |  |
| Outcomes | | | | | |
| All-cause mortality, n (%) | | | | | <0.01 |
| YES | 1260 (23.19) | 382 (21.09) | 385 (21.26) | 493 (27.22) |  |
| NO | 4173 (76.81) | 1429 (78.91) | 1426 (78.74) | 1318 (72.78) |  |
| Cardiovascular mortality, n (%) | | | | | <0.01 |
| YES | 337 (6.20) | 99 (5.47) | 99 (5.47) | 139 (7.68) |  |
| NO | 5096 (93.80) | 1712 (94.53) | 1712 (94.53) | 1672 (92.32) |  |
| Follow-up time (months) | 69.00 [37.00, 108.00] | 72.00 [38.00,108.00] | 71.00 [38.00,108.50] | 67.00 [36.00,105.00] | 0.181 |
| Medical history | | | | | |
| Drinking, n (%) | 3726 (68.58) | 1101 (60.80) | 1251 (69.08) | 1374 (75.87) | <0.01 |
| Smoking, n (%) | 2753 (50.67) | 728 (40.20) | 946 (52.24) | 1079 (59.58) | <0.01 |
| Diabetes, n (%) | 1854 (34.12) | 426 (23.52) | 614 (33.90) | 814 (44.95) | <0.01 |
| Hypercholesterolemia, n (%) | 3478 (64.02) | 1100 (60.74) | 1209 (66.76) | 1169 (64.55) | <0.01 |
| HF, n (%) | 489 (9.00) | 107 (5.91) | 145 (8.01) | 237 (13.09) | <0.01 |
| CAD, n (%) | 701 (12.90) | 167 (9.22) | 249 (13.75) | 285 (15.74) | <0.01 |
| Angina, n (%) | 381 (7.01) | 91 (5.02) | 132 (7.29) | 158 (8.72) | <0.01 |
| Heart attack, n (%) | 630 (11.60) | 151 (8.34) | 213 (11.76) | 266 (14.69) | <0.01 |
| Stroke, n (%) | 553 (10.18) | 188 (10.38) | 185 (10.22) | 180 (9.94) | 0.906 |
| Physical examination | | | | | |
| Weight (kg) | 78.60 [67.70, 91.70] | 63.80 [57.60,68.95] | 78.60 [73.60,83.90] | 98.10 [89.95,109.05] | <0.01 |
| Height (cm) | 164.40 [157.30, 171.90] | 158.80 [153.50,164.70] | 165.60 [158.70,171.50] | 170.30 [162.50,177.00] | <0.01 |
| BMI (kg/m^2^) | 28.90 [25.70, 33.00] | 24.90 [22.70,27.10] | 28.70 [26.70,31.14] | 34.20 [31.16,38.40] | <0.01 |
| WC (cm) | 102.80 [94.30, 112.20] | 90.70 [85.20,94.70] | 102.90 [100.00,105.70] | 117.20 [112.20,124.70] | <0.01 |
| Inflammatory indexes | | | | | |
| SIRI, | 1.03 [0.69,1.58] | 1.19 [0.80,1.80] | 1.31 [0.89,1.97] | 1.03 [0.69,1.58] | <0.01 |
| MHR | 0.35 [0.25,0.48] | 0.44 [0.32,0.59] | 0.50 [0.37,0.66] | 0.35 [0.25,0.48] | <0.01 |
| NLR | 2.00 [1.44,2.80] | 2.09 [1.56,2.92] | 2.23 [1.62,3.00] | 2.00 [1.44,2.80] | <0.01 |
| Blood cell count | | | | | |
| Leukocyte, 10^9^/L | 6.90 [5.70, 8.30] | 6.50 [5.40,7.80] | 6.90 [5.70,8.20] | 7.30 [6.10,8.70] | <0.01 |
| Lymphocyte, 10^9^/L | 1.90 [1.50, 2.40] | 1.80 [1.50,2.40] | 1.90 [1.50,2.40] | 2.00 [1.50,2.50] | <0.01 |
| Monocyte, 10^9^/L | 0.60 [0.40, 0.70] | 0.50 [0.40,0.60] | 0.60 [0.50,0.70] | 0.60 [0.50,0.70] | <0.01 |
| Neutrophils, 10^9^/L | 4.00 [3.20, 5.20] | 3.70 [2.90,4.70] | 4.00 [3.20,5.10] | 4.40 [3.40,5.50] | <0.01 |
| Platelet, 10^9^/L | 229.00 [191.00, 272.00] | 233.00 [196.00,278.00] | 228.00 [191.00,271.00] | 226.00 [186.00,268.00] | <0.01 |
| Laboratory examination | | | | | |
| ALT (U/L) | 21.00 [17.00, 26.00] | 20.00 [16.00,25.00] | 21.00 [17.00,26.00] | 22.00 (17.00,27.00] | <0.01 |
| AST (U/L) | 22.00 [18.00, 27.00] | 22.00 [18.00,26.00] | 22.00 [18.00,27.00] | 22.00 [18.00,28.00] | 0.042 |
| Albumin (g/L) | 42.00 [40.00, 44.00] | 42.00 [40.00,44.00] | 42.00 [40.00,44.00] | 41.00 [39.00,43.00] | <0.01 |
| Bun (mg/mL) | 16.00 [13.00, 20.00] | 16.00 [12.00,20.00] | 16.00 [13.00,20.00] | 16.00 [13.00,21.00] | <0.01 |
| Creatinine (umol/L) | 83.98 [69.84, 102.54] | 78.68 [64.53,95.47] | 83.98 [70.72,103.43] | 88.40 [73.37,106.96] | <0.01 |
| UA (umol/L) | 345.00 [291.50, 404.50] | 315.20 [261.70,368.80] | 345.00 [291.50,410.40] | 368.80 [315.20,428.30] | <0.01 |
| Glucose (mmol/L) | 5.61 [5.05, 6.66] | 5.38 [4.94,6.11] | 5.61 [5.05,6.66] | 5.94 [5.22,7.27] | <0.01 |
| Cholesterol (mmol/L) | 4.76 [4.03, 5.53] | 4.99 [4.27,5.71] | 4.73 [4.03,5.53] | 4.50 [3.85,5.33] | <0.01 |
| Triglyceride (mmol/L) | 1.51 [1.03, 2.22] | 1.32 [0.90,1.94] | 1.52 [1.04,2.22] | 1.69 [1.19,2.46] | <0.01 |
| HDL (mmol/L) | 1.32 [1.09, 1.63] | 1.50 [1.24,1.81] | 1.29 [1.08,1.58] | 1.19 [1.01,1.40] | <0.01 |
| HbA1c (%) | 5.90 [5.60, 6.40] | 5.80 [5.50,6.10] | 5.90 [5.50,6.40] | 6.10 [5.70,6.80] | <0.01 |

*WC* waist circumference, *AVI* abdominal volume index, *PIR* poverty-to-income ratio, *BMI* body mass index, *NLR* neutrophil to lymphocyte ratio, *SIRI* systemic inflammatory response index, *MHR* monocyte to high density lipoprotein, *CAD*coronary artery disease, *HF* heart failure, *HDL* high-density lipoprotein cholesterol, *BUN* blood urea nitrogen, *UA* uric acid, *ALT* alanine aminotransferase, *AST* aspartate aminotransferase, HBA*1c* hemoglobin A1c.

**Table 2 The associations of AVI with all-cause mortality and cardiovascular mortality**

|  | **HR (95% CI)** | | |
| --- | --- | --- | --- |
|  | **Model 1** | **Model 2** | **Model 3** |
| All-cause mortality | | | |
| AVI | 1.03 (1.02, 1.04)** | 1.04 (1.03, 1.05)** | 1.09 (1.06,1.11)** |
| Tertiles 1 | 1 | 1 | 1 |
| Tertiles 2 | 1.01 (0.88, 1.16) | 0.99 (0.86, 1.15) | 0.98 (0.83, 1.15) |
| Tertiles 3 | 1.34 (1.17, 1.53)** | 1.38 (1.19, 1.59)** | 1.36 (1.10, 1.68)** |
| Cardiovascular mortality | | | |
| AVI | 1.04 (1.02, 1.06)** | 1.06 (1.03, 1.08)** | 1.07 (1.03, 1.12)** |
| Tertiles 1 | 1 | 1 | 1 |
| Tertiles 2 | 1.00 (0.76, 1.32) | 0.97 (0.73, 1.29) | 0.85 (0.63, 1.16) |
| Tertiles 3 | 1.46 (1.13, 1.89)** | 1.48 (1.12, 1.96)** | 1.10 (0.74, 1.64) |

Model 1 was unadjusted.

Model 2 was adjusted for gender, age, race, and education level.

Model 3 was additionally modified to account for PIR, BMI, smoking, drinking, diabetes, hypercholesterolemia, cholesterol, triglycerides.

*AVI* abdominal volume index*, BMI* body mass index, *PIR* Ratio of family income to poverty.

*HR* hazard ratio, *CI* confidence interval.

**Table 3 The associations between AVI and inflammatory parameters**

|  | **OR** | **95% CI** | **P value** |
| --- | --- | --- | --- |
| SIRI | | | |
| Model 1 | 1.12 | 1.07, 1.18 | <0.01 |
| Model 2 | 1.08 | 1.02, 1.13 | <0.01 |
| Model 3 | 1.05 | 0.98, 1.13 | =0.151 |
| MHR | | | |
| Model 1 | 5.69 | 4.47, 7.24 | <0.01 |
| Model 2 | 3.72 | 2.90, 4.76 | <0.01 |
| Model 3 | 1.58 | 1.15, 2.17 | <0.01 |
| NLR |  |  |  |
| Model 1 | 1.04 | 1.01, 1.08 | =0.016 |
| Model 2 | 1.02 | 0.99, 1.06 | =0.23 |
| Model 3 | 1.05 | 0.99, 1.11 | =0.092 |

Model 1 was unadjusted.

Model 2 was adjusted for gender, age, race, and education level.

Model 3 was additionally modified to account for PIR, BMI, smoking, drinking, diabetes, hypercholesterolemia, cholesterol, triglycerides, *NLR* neutrophil to lymphocyte ratio, *SIRI* systemic inflammatory response index, *MHR* monocyte to high density lipoprotein.

*AVI* abdominal volume index*, BMI* body mass index, *PIR* Ratio of family income to poverty.

*OR* odds ratio, *CI* confidence interval.

**Table 4 The associations of inflammatory parameters with all-cause mortality and cardiovascular mortality**

|  | **HR (95% CI)** | | |
| --- | --- | --- | --- |
|  | **Model 1** | **Model 2** | **Model 3** |
| All-cause mortality | | | |
| SIRI | 1.23 (1.21, 1.25) | 1.19 (1.16, 1.21) | 1.19 (1.16, 1.22) |
| MHR | 1.25 (1.18, 1.32) | 1.16 (1.10, 1.24) | 1.14 (1.07, 1.21) |
| NLR | 1.14 (1.12, 1.15) | 1.11 (1.09, 1.13) | 1.11 (1.09, 1.13) |
| Cardiovascular mortality | | | |
| SIRI | 1.24 (1.20, 1.28) | 1.20 (1.15, 1.25) | 1.20 (1.15, 1.25) |
| MHR | 1.25 (1.12, 1.39) | 1.14 (1.01, 1.29)* | 1.12 (0.98, 1.27)& |
| NLR | 1.15 (1.12, 1.18) | 1.12 (1.08, 1.15) | 1.12 (1.08, 1.16) |

Model 1 was unadjusted.

Model 2 was adjusted for gender, age, race, and education level.

Model 3 was additionally modified to account for PIR, BMI, smoking, drinking, diabetes, hypercholesterolemia, cholesterol, triglycerides, *NLR* neutrophil to lymphocyte ratio, *SIRI* systemic inflammatory response index, *MHR* monocyte to high density lipoprotein.

*AVI* abdominal volume index, *BMI* body mass index, *PIR* Ratio of family income to poverty.

*HR* hazard ratio, *CI* confidence interval.
