# Supplementary material for: The association of lipid accumulation product with inflammatory parameters and mortality: evidence from a large population-based study
Source: Front Epidemiol. 2025 Feb 4;4:1503261. doi: 10.3389/fepid.2024.1503261 (PMC11832662; doi:10.3389/fepid.2024.1503261)
Supplement: Supplementary file 2 [file Supplementaryfile1.pdf]

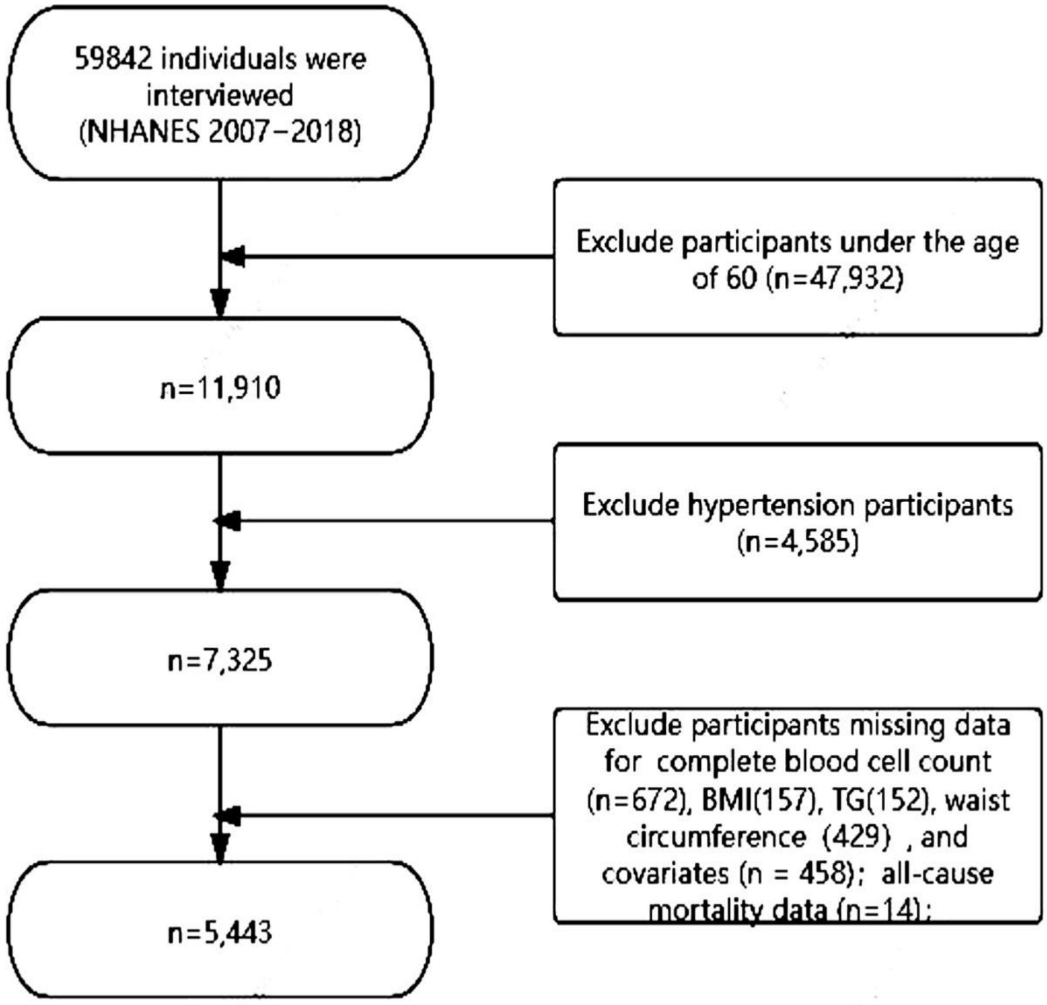

Figure 1 Flow diagram of the selection of eligible participants

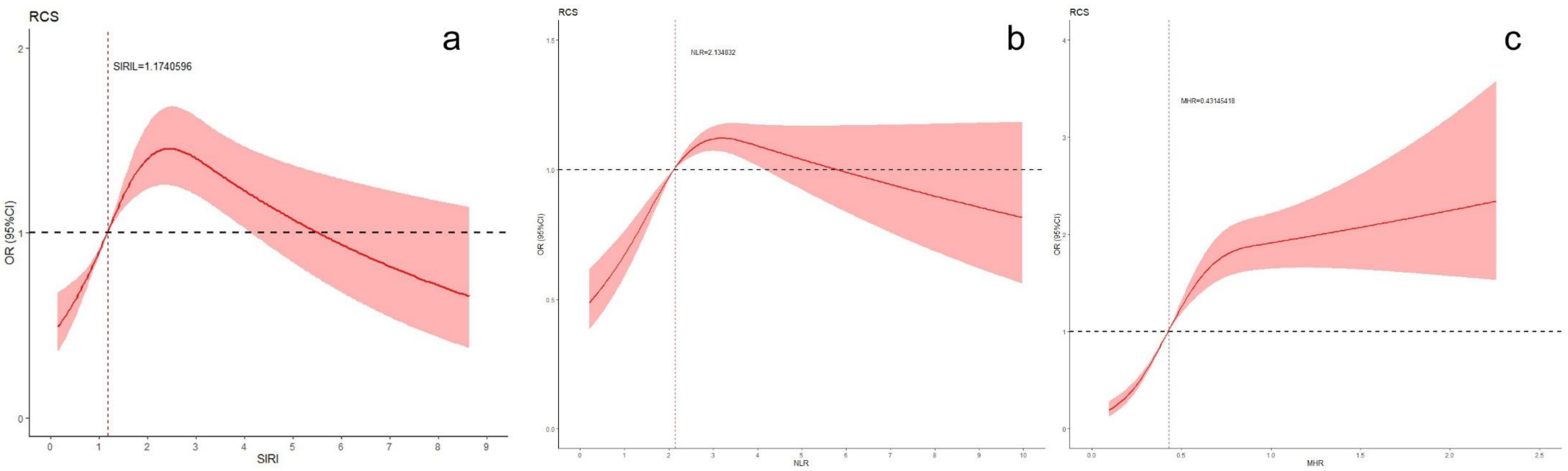

Figure 2 (a) The restricted cubic spline regression curve of the association between abdominal volume index (AVI) and systemic inflammatory response index (SIRI). **(b)** The restricted cubic spline regression curve of the association between AVI and neutrophil to neutrophil to lymphocyte ratio (NLR). **(c)** The restricted cubic spline regression curve of the association between AVI and monocyte to high density lipoprotein (MHR). The value corresponding to the red vertical line is the cutoff value in the x-axis.

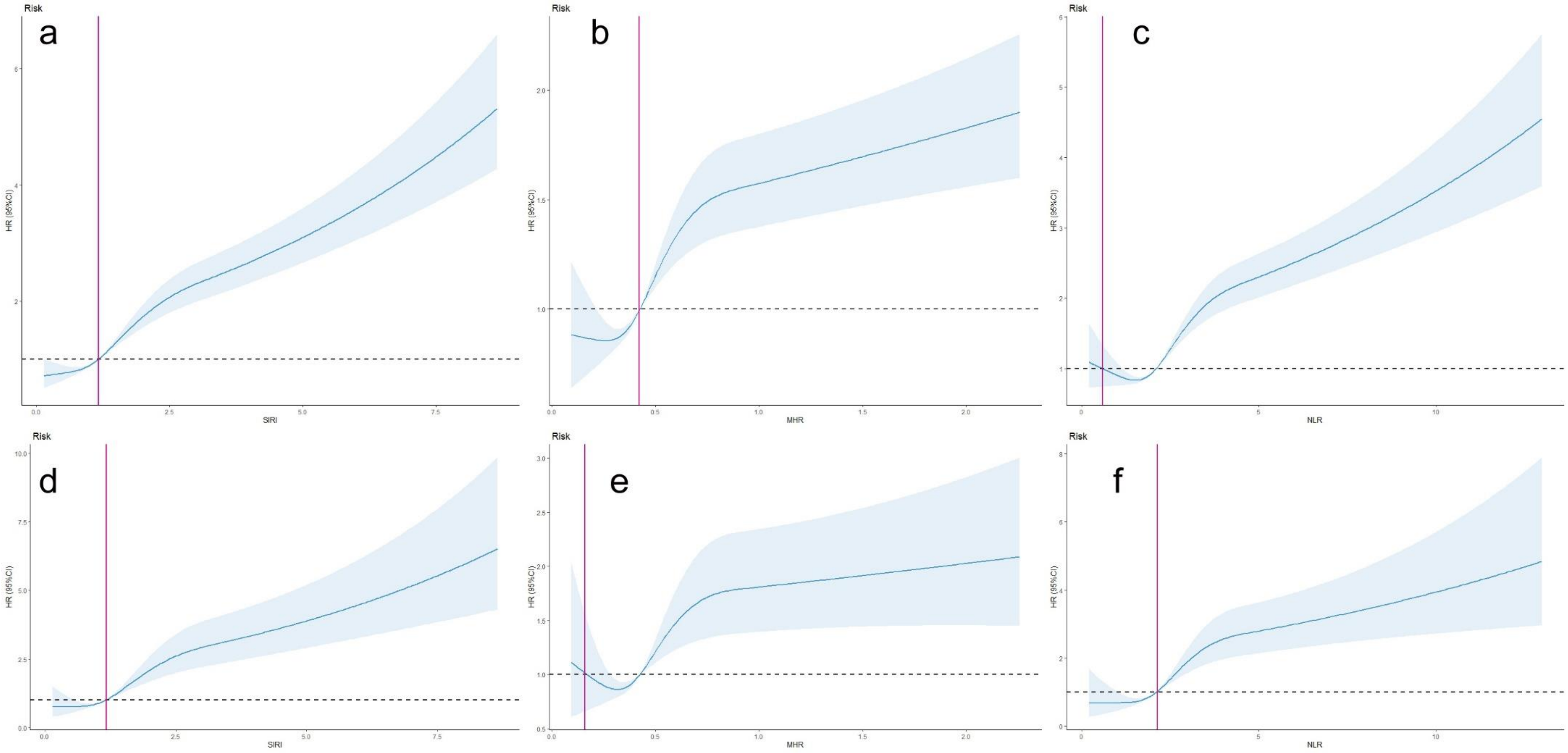

Figure 3 The restricted cubic spline regression curve of the association between mortality and inflammatary parameters. (**a**) systemic inflammatory response index (SIRI) and all-cause mortality. (**b**) monocyte to high density lipoprotein (MHR) and all-cause mortality. (**c**) neutrophil to lymphocyte ratio (NLR) and all-cause mortality. (**d**) SIRI and cadiovscular mortality. (**e**) MHR and cadiovscular mortality. (**f**) NLR and cadiovscular mortality.

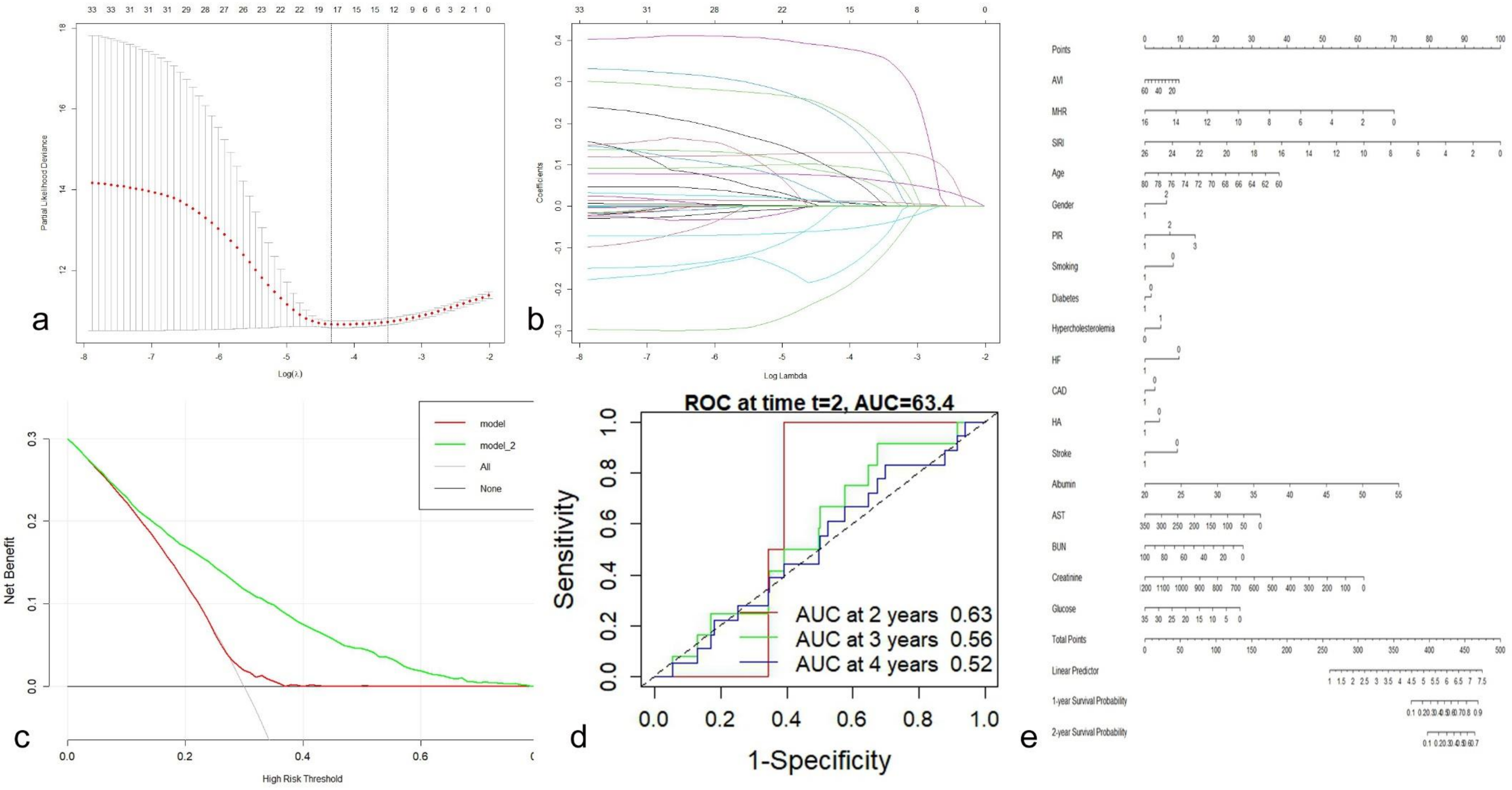

Figure 4 Establishment and validation of a risk prediction model for mortality based on abdominal volume index (AVI). **(a)** The coefficient shrinkage process of all 33 covariates. we represent the changes in coefficients of different features under

various levels of shrinkage by drawing lines of different colors. **(b)** A 10-fold cross-validation of the LASSO regression model. LASSO, least absolute shrinkage and selection operator. **(c)** Decision Curve Analysis (DCA) is a tool for evaluating the role of models in predicting mortality risk in AVI. **(d)** receiver operating characteristic (ROC) curve for evaluating the predictive power for mortality of the nomogram model. **(e)** A nomogram model based on AVI, systemic immune-inflammation index (SIRI), monocyte to high density lipoprotein (MHR), age, gender, poverty-to-income ratio (PIR), smoking, diabetes, hypercholesterolemia, heart failure (HF), coronary heart disease (CAD), heart attack (HA), stroke, albumin, aspartate aminotransferase (AST), blood urea nitrogen (BUN), creatinine, and glucose identified by LASSO regression analysis.
